# Tenotomy or Tenodesis for the Long Head of Biceps Lesions in Shoulders: A Systematic Review and Meta-Analysis

**DOI:** 10.1371/journal.pone.0121286

**Published:** 2015-03-18

**Authors:** Heng’an Ge, Qiang Zhang, Yeqing Sun, Jie Li, Lin Sun, Biao Cheng

**Affiliations:** 1 Department of Orthopedics, Shanghai Tenth People's Hospital, Tongji University School of Medicine, Shanghai, China; 2 Department of Orthopedics, Changzhou No.2 People's Hospital, Nanjing Medical University, Changzhou, China; Queen Mary University of London, UNITED KINGDOM

## Abstract

**Background:**

Both tenotomy and tenodesis have been widely used for the treatment of long head of biceps tendon (LHBT) lesions, but the optimal strategy remains considerably controversial. In this meta-analysis of published studies, we compared the results of the two procedures.

**Methods:**

A literature search that compared tenotomy with tenodesis was performed using MEDLINE, and Embase until August 2014. A total of 7 studies reporting data on 622 subjects were included. Study quality was evaluated using the PEDro critical appraisal tool and the NO quality assessment tool.

**Results:**

Data synthesis showed higher functional outcomes, a lower complication rate, and longer surgical time in patients managed with tenodesis compared to tenotomy (Constant score, *P* = 0.02; Popeye sign, *P* < 0.001; cramp pain, *P* = 0.04; surgical time, *P* < 0.001, respectively).

**Conclusion:**

This meta-analysis indicates that tenodesis results in better arm function and lower incidences of cramp pain and Popeye sign in LHBT lesions, while the procedure required longer surgical time compared to tenotomy. More sufficiently powered studies would be required to further determine the optimal strategy.

## Introduction

As a structure of the glenohumeral joint, the long head of biceps tendon (LHBT) originates from the glenoid labrum and the supraglenoid tubercle, runs in the bicipital groove, and exits via the intraarticular space [1]. The exact function of the LHBT in glenohumeral biomechanics is not clearly understood. Some researchers think that the LHBT has a role in humeral head depression and glenohumeral stabilization, whereas some others suggest that the tendon plays no role [2–5].

The LHBT lesion is a common cause of anterior shoulder pain with associated dysfunction of forward flexion [6,7]. The tendon lesion may be affected by numerous pathologic factors that can be broadly classified as inflammation, subluxation and traumatic lesions [8–10]. Usually, conservative management is preferred, including activity modification, rest, nonsteroidal anti-inflammatory drugs, physical therapy, and steroid injection [9,11–14]. If symptoms treated conservatively persist for longer than 3 months, surgical inventions are often indicated [6,9]. The surgical approach includes open and arthroscopic procedures [11]. Arthroscopic management has been recommended by an increasing number of surgeons [8,15,16]. The most common arthroscopic procedures for the treatment of LHBT lesions are tenotomy and tenodesis [11].

Arthroscopic biceps tenotomy is an easy and well-tolerated procedure that decreases operative time, simplifies postoperative rehabilitation, and allows patients return to activity as soon as possible [13,14,17]. Nonetheless, the procedure has its drawbacks, including the possible deformity of the anatomic profile of the arm (Popeye sign), possible cramping and fatigue pain, and biomechanical changes to the effects of LHBT capability on the humeral head [7,18–20]. As a result of the disadvantages associated with tenotomy, some surgeons advocate that tenodesis results in a better ability to return to activity, a decreased rate of cosmetic deformities and cramping pain, and a closer approximation of normal anatomy in spite of longer rehabilitation times and a more technically demanding surgery [18,21–23].

While both tenotomy or tenodesis can produce favorable results in LHBT lesions, no agreement has been reached regarding the superiority of either technique [24]. Numerous studies [1,8,10,16,25–27] have compared the outcomes of biceps tenotomy and the various tenodesis techniques in the treatment of LHB lesions, but the optimal surgical strategy remains controversial. Moreover, there has been no published meta-analysis comparing the two treatment procedures. Therefore, to determine which of the two methods can produce better clinical and functional outcomes, we performed this meta-analysis based on the relevant available online literature.

## Materials and Methods

### Search strategy

Two independent researchers reviewed the PubMed/MEDLINE and EMBASE databases for eligible clinical articles published between July 1967 and August 2014, utilizing the keywords “long head of biceps”, “biceps tenotomy”, and “biceps tenodesis”. Relevant articles in reference lists of published articles were also searched. The search was performed in August 2014.

### Inclusion and Exclusion criteria

All studies in our review were selected according to the following criteria: 1) patients diagnosed with LBHT lesions; 2) patients failed conservative treatment for more than 3 months; 3) comparison of the arthroscopic tenotomy and tenodesis; 4) patient outcomes of the two treatment techniques; and 5) more than a 1 year follow-up period available. In cases of missing data, or when mean or standard deviation (SD) values were not presented, the corresponding author(s) were contacted via e-mail for more relevant information, if necessary. Studies were excluded if 1) the studies were not published in English or were case reports or reviews; 2) studies were animal experiments or in vitro trials; 3) follow-up was less than 1 year; and 4) if the missing relevant information in the studies was difficult to acquire or if it was impossible to extrapolate or calculate the necessary data from the published results.

### Data extraction and Methodological quality assessment

Two independent reviewers extracted all data from the full-text versions of eligible studies. The extracted information included 1) the first author, year of publication, country of author, study type and study duration; 2) the number and characteristics of subjects; 3) concomitant injuries; and 4) outcomes. Discrepancies were resolved by consensus first and then were eventually determined by a third reviewer. Once completed, all data were synthesized into an agreed-upon data extraction table and prepared for data analysis.

Study quality was independently assessed by two reviewers. The Physiotherapy Evidence Database (PEDro) scale [28] was used to evaluate the risk of bias for randomized controlled trials (RCT) evidence. The quality of each non-randomized study was assessed using the Newcastle–Ottawa (NO) quality assessment tool [29]. The NO scale is based on standard quality ratings as follows: 1) selection of study groups; 2) comparability of study groups; and 3) ascertainment of either the exposure (case control) or the outcome of interest (cohort study).

### Outcome measurement

We defined the primary outcome measures in this review as functional scores and range of motion (ROM). The functional scores included University of California-Los Angeles (UCLA) Scores, Constant score, and visual analog score (VAS). ROM included forward flexion and external rotation. Secondary outcome measures under investigation included the incidence of Popeye sign and cramping pain, degree of patient satisfaction, and surgical time.

### Statistical analysis

The data were pooled using Review Manager 5.2 software. Analysis of the treatment effect was performed when no substantial differences in study populations, interventions or outcome measurements were observed. For each study, we calculated a risk ratio (RR) with 95% confidence intervals (CIs) for dichotomous data and mean differences (MD) with 95% CIs for continuous data. Heterogeneity was tested using the chi-squared and I^2^ statistics. Studies with an I^2^ statistic of > 50% were considered to have substantial heterogeneity, and therefore, a random effects model analysis was used. Otherwise, a fixed-effect model was initially employed in the analysis. A *P* value lower than 0.05 or a 95% CI that did not contain unity was considered statistically significant. A Forest plot was utilized for the presentation of summarized outcomes. Publication bias was not examined due to the small number of studies (< 10) included in each analysis.

## Results

### Search results

**Fig 1:**
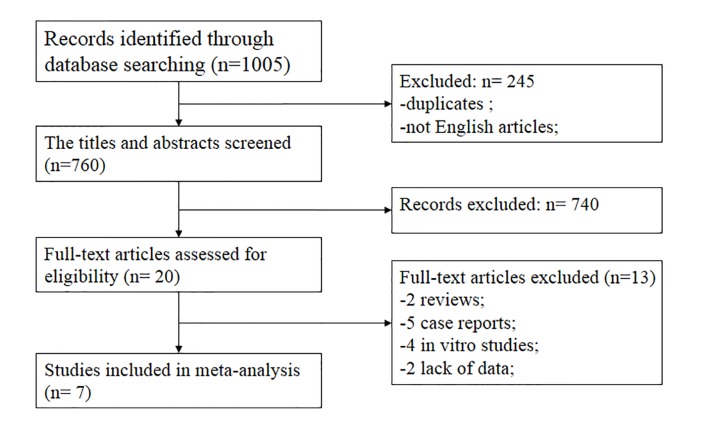
Flow diagram of study selection.

The search of the online database yielded 1005 publications. We screened abstracts for those that compared tenotomy and tenodesis for LHBT lesions and identified 20 articles that met the inclusion criteria. Finally, seven articles [1,8,10,16,25–27] were included in this study based on the inclusion/exclusion criteria after full-text reviews. Three of the included studies were RCTs, and another 4 studies were cohort studies. The flow diagram of the study search process is presented in Fig. 1. The general characteristics of the included studies are provided in Tables 1 and 2. With respect to the concomitant injuries, rotator cuff repair, acromioplasty, and distal clavicle resection were performed, except in the study of Boileau et al [27]. The mean PEDro score of the 7 trials was 8.0 (SD = 0.82); the detailed results are summarized in Table 3. Table 4 represents the quality of the four cohort studies, as determined using the NO scale. Both the studies of Rose et al [1] and Boileau et al [27] were of low quality. Because neither Rose et al [1] nor Boileau et al [27] included the patients based on age, gender and other factors, the poor comparability was displayed.

**Table 1:** Characteristics of studies included in this meta-analysis.

References	Year	Country	Study design	Duration (m)	
				Td	Tt
Franceschi et al [25]	2007	UK	RCT	62.4	62.4
Carli et al [26]	2012	Italy	RCT	24.5	22.2
Zhang et al [10]	2013	China	RCT	24.7	24.7
Koh et al [8]	2010	Korea	Cohort	27.9	27.1
Rose et al [1]	2012	Italy	Cohort	51.6	51.6
Cho et al [16]	2014	Korea	Cohort	26.1	24.2
Boileau et al [27]	2007	France	Cohort	36	34

**Abbreviations**: RCT, Randomized controlled trial; Td, Tenodesis; Tt, Tenotomy.

**Table 2:** Patient characteristics of the included studies.

References	Shoulders		Mean age (y)		Gender (m/f)		Dm/Ndm, No.		concomitant injuries, No.	
	Td	Tt	Td	Tt	Td	Tt	Td	Tt	Td	Tt
Franceschi et al [25]	31	32	61.8	64.7	18/13	15/17	25/6	23/9	RCR, 31; Am, 7.	RCR, 32; Am, 9;
Carli et al [26]	35	30	56.3	59.6	48/17	48/17	NR	NR	RCR, 35;	RCR, 30;
Zhang et al [10]	74	77	62	61	35/39	36/41	NR	NR	RCR, 74; Am, 14; DCR, 5;	RCR, 77; Am, 17; DCR, 6;
Koh et al [8]	43	41	61	66	16/27	9/32	NR	NR	RCR, 43; Am, 2; DCR, 5;	RCR, 41; Am, 6; DCR, 3;
Rose et al [1]	56	48	45.6	51.4	24/32	22/26	36/20	37/11	Only LHB;	Only LHB;
Cho et al [16]	42	41	58.6	63.8	23/19	20/21	24/18	28/13	RCR, 42;	RCR, 41;
Boileau et al [27]	33	39	68	68	19/14	9/30	63/9	63/9	CILU;	CILU;

**Abbreviations**: Dm, Dominant; Ndm, Nondominant; Td, Tenodesis; Tt, Tenotomy; RCR, Rotator cuff repair; Am, Acromioplasty; DCR, Distal clavicle resection; LHB, Long head of the biceps; CILU, Concomitant injuries left untreated; NR, Not reported.

**Table 3:** RCTs quality ratings (determined using the PEDro critical appraisal score).

	Franceschi [25]	Carli [26]	Zhang [10]
Eligibility criteria	Yes	Yes	Yes
Random allocation	Yes	Yes	Yes
Concealed allocation	Yes	Yes	Yes
Baseline comparability	Yes	Yes	Yes
Blind subject	Yes	Yes	Yes
Blind clinician	No	No	Yes
Blind assessor	No	No	Yes
Adequate follow-up	Yes	Yes	Yes
Intention-to treat analysis	Yes	Yes	Yes
Between-group analysis	Yes	Yes	Yes
Point estimates and variability	Yes	Yes	Yes
Total score	8	7	9

**Table 4:** Cohort study quality rating (determined using the Newcastle–Ottawa scale).

Study	Year	Selection	Comparability	Outcome
Koh [8]	2010	****	**	***
Rose [1]	2012	***	-	**
Cho [16]	2014	****	**	***
Boileau [27]	2007	**	-	**

**Assessment strategies**: selection (max. 4 stars), comparability (max. 2 stars), and outcome (max. 3 stars).

### Results of pooled analysis

**Fig 2:**
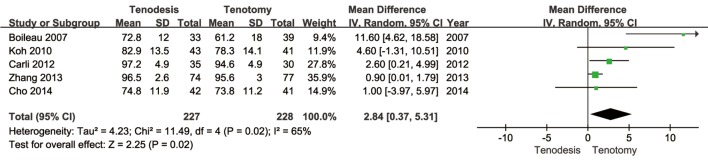
Forest plot to assess Constant score between two treatment strategies.

Table 5 presents the evaluation data which could be performed meta-analysis due to the lack of standard deviation. Finally, only Constant score in primary outcomes groups could be conducted meta-analysis. For the Constant score, one study [1] did not provide the standard deviation, so a meta-analysis could only be performed with five studies. Because significant heterogeneity was observed among the two groups (*P* = 0.02; I^2^ = 65%), a random effect model was employed. The overall pooled results of five studies [8,10,16,26,27] revealed a significantly higher result for tenodesis as compared to tenotomy group (*P* = 0.02, Fig. 2). Due to lack of complete data, no meta-analysis could be performed for ROM. The evaluation of ROM was performed in three studies [16,25,27]. All of the three studies reported no statistical difference between the two treatments, with regarding to forward flexion, external rotation, and internal rotation.

**Table 5:** Physical examination rating scales (meta-analysis not performed).

	UCLA Scores		Constant score		VAS		Forward flexion		External rotation		Internal rotation	
	Td	Tt	Td	Tt	Td	Tt	Td	Tt	Td	Tt	Td	Tt
Franceschi et al [25]	27.9 (24–35)	32.1(30–35)	-	-	-	-	139° (120°-170°)	166° (140°-170°)	121.4° (90°-140°)	134.3° (90°-140°)	34.3° (26°-40°)	40.0° (30°-40°)
Zhang et al [10]	-	-	-	-	2.1 ± 1.6	2.0 ± 1.1	-	-	-	-	-	-
Rose et al [1]	-	-	84.9 (51–98)	86.1 (53–100)	1.4 (0–5)	1.5 (0–6)	-	-	-	-	-	-
Cho et al [16]	31.3 ± 3.0	30.6 ± 4.1	-	-	0.3	0.2	154.1	156.4	53.3	53.9	T11.1	T11.9
Boileau et al [27]	-	-	-	-	-	-	173 ± 10.5	166.4 ± 21.3	52.3 ± 16.9	51.3 ± 16.8	L3	L3

**Abbreviations**: UCLA, University of California-Los Angeles; VAS, Visual Analog Score; Td, Tenodesis; Tt, Tenotomy;

**Fig 3:**
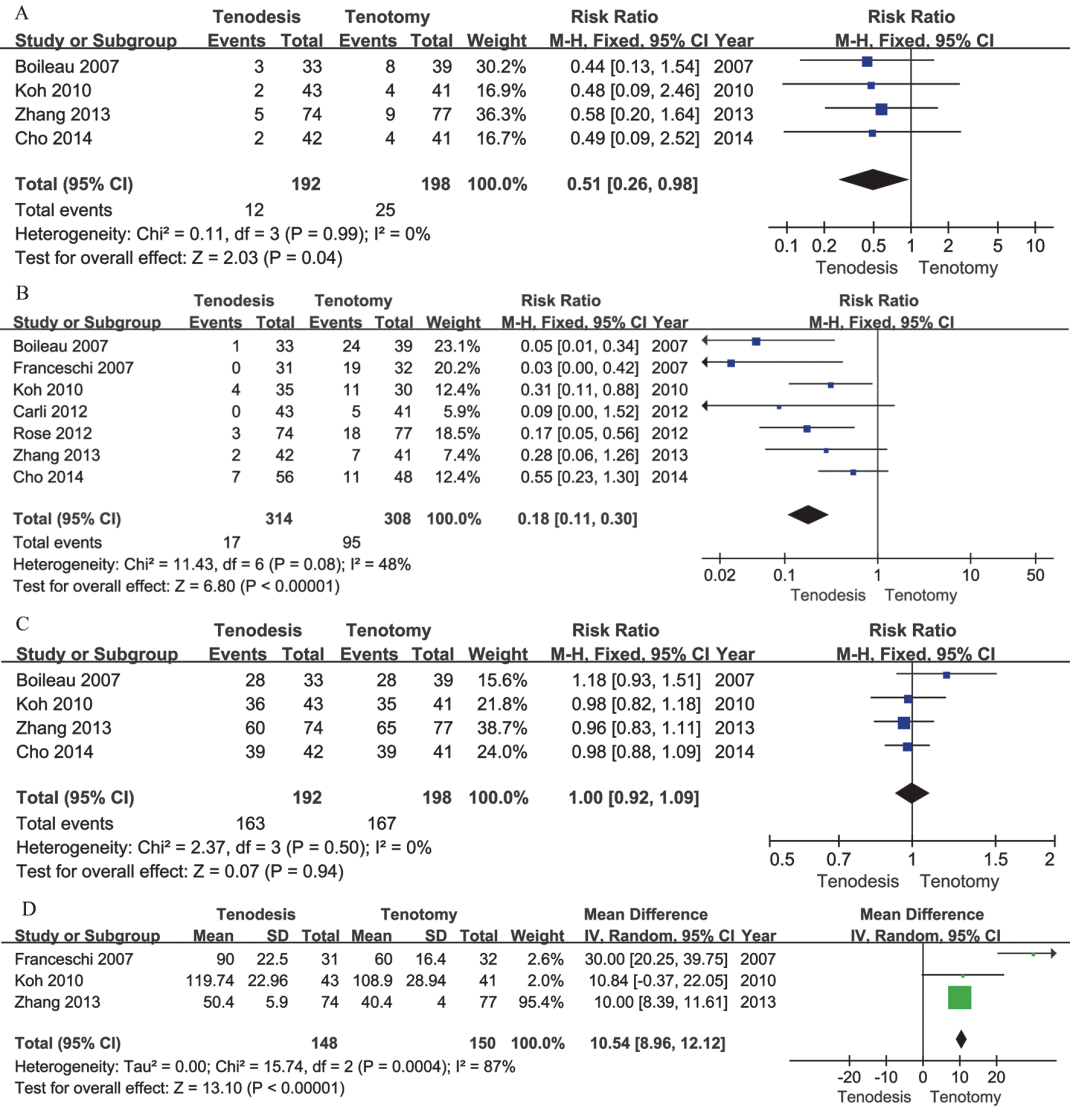
Secondary outcomes after meta-analysis. A Forest plot to assess cramps pain events between two treatment strategies. B Forest plot to assess Popeye sign events between two treatment strategies. C Forest plot to assess patient’s satisfaction events between two treatment strategies. D Forest plot to assess surgical time events between two treatment strategies.

Fig. 3 shows the pooled analysis of secondary outcomes. Regarding postoperative complications, the incidence of cramp pain and the Popeye sign was significantly reduced in the tenodesis group compared to the tenotomy group (*P* = 0.04, Fig. 3A; and *P* < 0.001, Fig. 3B, respectively). Four studies reported the degree of patient satisfaction [8,10,16,27], which was similar in the two groups (*P* = 0.94, Fig. 3C). Moreover, we also observed a significantly shorter surgical time in the tenotomy group (P < 0.001, Fig. 3D) compared to the tenodesis group, with significant heterogeneity (*P* < 0.001, I^2^ = 87%).

### Subgroup analysis

Subgroup analysis according to the type of tenodesis was available for the incidence of Popeye sign (Table 6). The type of tenodesis was divided into two subgroups (proximal groove tenodesis and soft tissue tenodesis). Both proximal groove tenodesis and soft tissue tenodesis could decrease the incidence of the Popeye sign, compared with the tenotomy procedure, but there was a moderate heterogeneity in proximal groove tenodesis subgroup (*I* ^2^ = 53%). Due to the potential relationship between chronic pathologies of the supraspinatus tendon and the LHBT described by Redondo-Alonso et al. [30], we also performed subgroup analysis based on the status of rotator cuff. Six [8,10,16,25–27] out of all the seven studies included the patients with rotator cuff tears and only one [1] included patients with isolated LHBT lesions. After meta-analysis, a statistically significant difference was found in patients with rotator cuff tears between the two treatments, with a moderate degree of heterogeneity.

**Table 6:** Subgroup analysis in Popeye sign according to the type of tenodesis and the status of rotator cuff.

Variables	Study	RRE (95%CI)	*I* ^2^	*P* value
Tenodesis				
Proximal groove tenodesis	[8, 10, 25, 27]	0.14[0.04, 0.48]	53%	0.002
Soft tissue tenodesis	[1, 16, 26]	0.29[0.15, 0.55]	43%	< 0.001
Rotator cuff				
Rotator cuff tears	[8, 10, 16, 25–27]	0.20[0.08, 0.51]	56%	< 0.001
Normal rotator cuff	[1]	N/A	N/A	0.004

**Abbreviations**: RRE, risk ratio effect; N/A, not applicable.

## Discussion

The most important finding of this systematic review and meta-analysis is that the patients with LHBT lesions undergoing the tenodesis procedure achieve higher functional scores, better ROM, and lower incidence of cramp pain and Popeye sign than those managed with the tenotomy strategy. Furthermore, the arthroscopic tenotomy strategy requires a shorter surgical time and achieves similar levels of postoperative pain and patient satisfaction compared with arthroscopic tenodesis. However, these findings should be treated with caution because only 3 RCTs were reviewed in this meta-analysis.

Lesions of the LHBT are often responsible for shoulder pain and dysfunction. The optimal treatment for these injuries remains debatable. Aiming to relieve pain and restore shoulder function, arthroscopic biceps tenotomy and tenodesis are two well-established surgical procedures [13,18,31]. A number of relevant studies show a reliable improvement in postoperative outcomes for patients with LHBT lesions treated with tenotomy, as well as with tenodesis. Although numerous studies have published biomechanical or clinical results comparing tenotomy and tenodesis strategies, few trials have definitively determined which one is superior. Therefore, we conducted this meta-analysis to systematically evaluate the clinical outcomes of treatment for LHBT lesions managed with biceps tenotomy versus tenodesis procedures.

In 2005, Walch et al. [20] reported the long-term clinical and radiological results in a group of 307 patients managed with biceps tenotomy. They found significant improvements in Constant scores, with 87% of patients satisfied by the surgical outcome after a mean of 57 months of follow-up. Elkousy et al. [32] presented good results for patients undergoing arthroscopic tenodesis in terms of pain relief, good function and lack of muscle deformity. None of the patients in that study reported the Popeye sign or cramp pain. Therefore, both tenotomy and tenodesis can effectively improve function in the affected arm. Both Osbahr et al. [33] and Edwards et al. [34] found no differences in outcomes between the 2 techniques. In a systematic review on LHBT lesions treated with tenotomy versus tenodesis, Frost et al. [17] reported no significant differences in subjective outcomes between the 2 procedures. However, Franceschi et al [35] found that patients undergoing tenotomy had significantly better results in shoulder function and higher satisfaction levels compared with those treated with tenodesis. The findings of our meta-analysis are not consistent with the results discussed above. However, our findings regarding functional outcomes should be interpreted with great caution, due to significant heterogeneity in the included studies. This might be because there was a lack of high-quality studies—this study only included three RCTs, the four included cohort studies were constrained by methodological deficiencies, and the period of follow-up in each included study varied. Nonetheless, data on patient satisfaction after meta-analysis showed no differences in our review. This may suggest that both procedures met the patients’ expectations. Nonetheless, more RCTs using standardized functional scores are needed in the future.

Regarding complications of the two procedures, the patients undergoing tenodesis rather than tenotomy tend to report lower incidences of cramp pain and Popeye sign. This is consistent with most reports in the literature. A systematic review of Slenker et al. [6] noted that a major difference between the 2 procedures was the increased occurrence of cosmetic deformity, or the Popeye sign, with tenotomy (42%) compared with tenodesis (8%). According to some studies, [13,23,25,36,37], there is a risk of Popeye sign (17–70%) and cramping pain (9–24%) in the patients treated with tenotomy. The results suggest that the tenodesis procedure should be widely applied to reduce complications. However, a web-based survey of 1,084 orthopedic surgeons found that most surgeons would choose tenotomy to treat a 65-year-old male who was diagnosed large rotator cuff tears and combined LHBT instability [38]. Because various limitations exist in both tenotomy and tenodesis procedures, the optimal treatment should be based on the severity of the disease and the physical conditions of the patients.

There are several limitations to this systematic review and meta-analysis. The strength of this meta-analysis is limited by a lack of high-level evidence on the comparison between tenotomy and tenodesis strategies. Four of the included studies were cohort studies, and the two assessed by the NO scale were of poor quality. Second, the sample sizes of the included studies were relatively small. Additionally, clinical heterogeneity may have been caused by the various indications for surgery and differences in surgical skill levels among the surgeons. Finally, the lack of a standard outcome measure by which to evaluate the postoperative clinical results may be responsible for the lack of convincing evidence.

## Conclusions

In this meta-analysis, better subjective outcomes and a lower incidence of cramp pain and the Popeye sign were observed in patients whose LHBT lesions were managed with the tenodesis procedure, whereas the arthroscopic tenotomy strategy was technically simpler and faster and had a similar degree of postoperative satisfaction. Due to the limited number and inherent methodological deficiencies of the available studies, future analysis of better-designed studies involving larger sample sizes will be required.

## Supporting Information

S1 Checklist (DOC)

## References

[1] Delle Rose G, Borroni M, Silvestro A, Garofalo R, Conti M, et al (2012) The long head of biceps as a source of pain in active population: tenotomy or tenodesis? A comparison of 2 case series with isolated lesions. Musculoskelet Surg 96 Suppl 1: S47–52. 10.1007/s12306-012-0189-0 22528844

[2] Rodosky MW, Harner CD, Fu FH (1994) The role of the long head of the biceps muscle and superior glenoid labrum in anterior stability of the shoulder. Am J Sports Med 22: 121–130. 8129095 10.1177/036354659402200119

[3] Yamaguchi K, Riew KD, Galatz LM, Syme JA, Neviaser RJ (1997) Biceps activity during shoulder motion: an electromyographic analysis. Clin Orthop Relat Res: 122–129. 10.1097/00003086-199703000-00017 9060495

[4] Kim SH, Ha KI, Kim HS, Kim SW (2001) Electromyographic activity of the biceps brachii muscle in shoulders with anterior instability. Arthroscopy 17: 864–868. 11600985 10.1016/s0749-8063(01)90011-8

[5] Itoi E, Kuechle DK, Newman SR, Morrey BF, An KN (1993) Stabilising function of the biceps in stable and unstable shoulders. J Bone Joint Surg Br 75: 546–550. 8331107 10.1302/0301-620X.75B4.8331107

[6] Slenker NR, Lawson K, Ciccotti MG, Dodson CC, Cohen SB (2012) Biceps tenotomy versus tenodesis: clinical outcomes. Arthroscopy 28: 576–582. 10.1016/j.arthro.2011.10.017 22284407

[7] Hsu AR, Ghodadra NS, Provencher MT, Lewis PB, Bach BR (2011) Biceps tenotomy versus tenodesis: a review of clinical outcomes and biomechanical results. J Shoulder Elbow Surg 20: 326–332. 10.1016/j.jse.2010.08.019 21051241

[8] Koh KH, Ahn JH, Kim SM, Yoo JC (2010) Treatment of biceps tendon lesions in the setting of rotator cuff tears: prospective cohort study of tenotomy versus tenodesis. Am J Sports Med 38: 1584–1590. 10.1177/0363546510364053 20551285

[9] Barber FA, Field LD, Ryu RK (2008) Biceps tendon and superior labrum injuries: decision making. Instr Course Lect 57: 527–538. 18399607

[10] Zhang Q, Zhou J, Ge H, Cheng B (2013) Tenotomy or tenodesis for long head biceps lesions in shoulders with reparable rotator cuff tears: a prospective randomised trial. Knee Surg Sports Traumatol Arthrosc. 10.1007/s00167-013-2587-8 23828089

[11] Ahrens PM, Boileau P (2007) The long head of biceps and associated tendinopathy. J Bone Joint Surg Br 89: 1001–1009. 17785735 10.1302/0301-620X.89B8.19278

[12] Sethi N, Wright R, Yamaguchi K (1999) Disorders of the long head of the biceps tendon. J Shoulder Elbow Surg 8: 644–654. 10633904 10.1016/s1058-2746(99)90105-2

[13] Gill TJ, McIrvin E, Mair SD, Hawkins RJ (2001) Results of biceps tenotomy for treatment of pathology of the long head of the biceps brachii. J Shoulder Elbow Surg 10: 247–249. 11408906 10.1067/mse.2001.114259

[14] Kelly AM, Drakos MC, Fealy S, Taylor SA, O'Brien SJ (2005) Arthroscopic release of the long head of the biceps tendon: functional outcome and clinical results. Am J Sports Med 33: 208–213. 15701606 10.1177/0363546504269555

[15] Desmarais S, Black WC, Oballa R, Lamontagne S, Riendeau D, et al (2008) Effect of cathepsin k inhibitor basicity on in vivo off-target activities. Mol Pharmacol 73: 147–156. 17940194 10.1124/mol.107.039511

[16] Cho NS, Cha SW, Rhee YG (2014) Funnel tenotomy versus intracuff tenodesis for lesions of the long head of the biceps tendon associated with rotator cuff tears. Am J Sports Med 42: 1161–1168. 10.1177/0363546514523719 24576743

[17] Frost A, Zafar MS, Maffulli N (2009) Tenotomy versus tenodesis in the management of pathologic lesions of the tendon of the long head of the biceps brachii. Am J Sports Med 37: 828–833. 10.1177/0363546508322179 18762669

[18] Elser F, Braun S, Dewing CB, Giphart JE, Millett PJ (2011) Anatomy, function, injuries, and treatment of the long head of the biceps brachii tendon. Arthroscopy 27: 581–592. 10.1016/j.arthro.2010.10.014 21444012

[19] Nassos JT, Chudik SC (2009) Arthroscopic rotator cuff repair with biceps tendon augmentation. Am J Orthop (Belle Mead NJ) 38: 279–281. 19649344

[20] Walch G, Edwards TB, Boulahia A, Nove-Josserand L, Neyton L, et al (2005) Arthroscopic tenotomy of the long head of the biceps in the treatment of rotator cuff tears: clinical and radiographic results of 307 cases. J Shoulder Elbow Surg 14: 238–246. 15889020 10.1016/j.jse.2004.07.008

[21] Berlemann U, Bayley I (1995) Tenodesis of the long head of biceps brachii in the painful shoulder: improving results in the long term. J Shoulder Elbow Surg 4: 429–435. 8665287 10.1016/s1058-2746(05)80034-5

[22] Boileau P, Krishnan SG, Coste JS, Walch G (2002) Arthroscopic biceps tenodesis: a new technique using bioabsorbable interference screw fixation. Arthroscopy 18: 1002–1012. 12426544 10.1053/jars.2002.36488

[23] Checchia SL, Doneux PS, Miyazaki AN, Silva LA, Fregoneze M, et al (2005) Biceps tenodesis associated with arthroscopic repair of rotator cuff tears. J Shoulder Elbow Surg 14: 138–144. 15789006 10.1016/j.jse.2004.07.013

[24] Miller T, Jones G, Moon Shoulder G (2011) Arthroscopic evaluation and treatment of biceps brachii long head tendon injuries: A survey of the MOON shoulder group. Int J Shoulder Surg 5: 68–71. 10.4103/0973-6042.86236 22058639 PMC3205525

[25] Franceschi F, Longo UG, Ruzzini L, Rizzello G, Maffulli N, et al (2008) No advantages in repairing a type II superior labrum anterior and posterior (SLAP) lesion when associated with rotator cuff repair in patients over age 50: a randomized controlled trial. Am J Sports Med 36: 247–253. 17940144 10.1177/0363546507308194

[26] De Carli A, Vadala A, Zanzotto E, Zampar G, Vetrano M, et al (2012) Reparable rotator cuff tears with concomitant long-head biceps lesions: tenotomy or tenotomy/tenodesis? Knee Surg Sports Traumatol Arthrosc 20: 2553–2558. 10.1007/s00167-012-1918-5 22349543

[27] Boileau P, Baque F, Valerio L, Ahrens P, Chuinard C, et al (2007) Isolated arthroscopic biceps tenotomy or tenodesis improves symptoms in patients with massive irreparable rotator cuff tears. J Bone Joint Surg Am 89: 747–757. 17403796 10.2106/JBJS.E.01097

[28] Maher CG, Sherrington C, Herbert RD, Moseley AM, Elkins M (2003) Reliability of the PEDro scale for rating quality of randomized controlled trials. Phys Ther 83: 713–721. 12882612

[29] Stang A (2010) Critical evaluation of the Newcastle-Ottawa scale for the assessment of the quality of nonrandomized studies in meta-analyses. Eur J Epidemiol 25: 603–605. 10.1007/s10654-010-9491-z 20652370

[30] Redondo-Alonso L, Chamorro-Moriana G, Jimenez-Rejano JJ, Lopez-Tarrida P, Ridao-Fernandez C (2014) Relationship between chronic pathologies of the supraspinatus tendon and the long head of the biceps tendon: systematic review. BMC Musculoskelet Disord 15: 377 10.1186/1471-2474-15-377 25408141 PMC4247626

[31] Su WR, Budoff JE, Luo ZP (2010) The effect of posterosuperior rotator cuff tears and biceps loading on glenohumeral translation. Arthroscopy 26: 578–586. 10.1016/j.arthro.2009.09.007 20434653

[32] Elkousy HA, Fluhme DJ, O'Connor DP, Rodosky MW (2005) Arthroscopic biceps tenodesis using the percutaneous, intra-articular trans-tendon technique: preliminary results. Orthopedics 28: 1316–1319. 16295187 10.3928/0147-7447-20051101-08

[33] Osbahr DC, Diamond AB, Speer KP (2002) The cosmetic appearance of the biceps muscle after long-head tenotomy versus tenodesis. Arthroscopy 18: 483–487. 11987057 10.1053/jars.2002.32233

[34] Edwards TB, Walch G, Sirveaux F, Mole D, Nove-Josserand L, et al (2006) Repair of tears of the subscapularis. Surgical technique. J Bone Joint Surg Am 88 Suppl 1 Pt 1: 1–10. 16510795 10.2106/JBJS.E.00842

[35] Franceschi F, Longo UG, Ruzzini L, Papalia R, Rizzello G, et al (2007) To detach the long head of the biceps tendon after tenodesis or not: outcome analysis at the 4-year follow-up of two different techniques. Int Orthop 31: 537–545. 16947053 10.1007/s00264-006-0206-8 PMC2267623

[36] Ahmad CS, DiSipio C, Lester J, Gardner TR, Levine WN, et al (2007) Factors affecting dropped biceps deformity after tenotomy of the long head of the biceps tendon. Arthroscopy 23: 537–541. 17478286 10.1016/j.arthro.2006.12.030

[37] Klepps S, Hazrati Y, Flatow E (2002) Arthroscopic biceps tenodesis. Arthroscopy 18: 1040–1045. 12426550 10.1053/jars.2002.36467

[38] Randelli P, Arrigoni P, Cabitza F, Ragone V, Cabitza P (2012) Current practice in shoulder pathology: results of a web-based survey among a community of 1,084 orthopedic surgeons. Knee Surg Sports Traumatol Arthrosc 20: 803–815. 10.1007/s00167-011-1673-z 21964496

